# Improved Fixation Stability of a Dedicated Rib Fixation System in Flail Chest: A Retrospective Study

**DOI:** 10.3390/medicina58030345

**Published:** 2022-02-24

**Authors:** Shang-Ting Tsai, Hung-Yu Lin, Chia-Ying Li, Chih-Chien Lin

**Affiliations:** 1 Department of Emergency Medicine, Show Chwan Memorial Hospital, Changhua 500, Taiwan; tim502323@gmail.com; 2 Research Assistant Center, Show Chwan Memorial Hospital, Changhua 500, Taiwan; linhungyu700218@gmail.com; 3 Department of Surgery, Show Chwan Memorial Hospital, Changhua 500, Taiwan; 4 Graduate Institute of Biomedical Engineering, National Chung Hsing University, Taichung 402, Taiwan

**Keywords:** flail chest, rib fracture, pre-contoured rib plate, fixation stability

## Abstract

*Background and Objectives*: Flail chest typically results from major trauma to the thoracic cage and is accompanied by multiple rib fractures. It has been well documented that surgical fixation of rib fractures can decrease both morbidity and mortality rates. This study aimed to evaluate the effectiveness of a dedicated APS Rib Fixation System, which features a pre-contoured design based on anatomical rib data of the Asian population. *Materials and Methods*: We reviewed 43 consecutive patients, who underwent surgical stabilization for flail chest with the traditional Mini bone plate (*n* = 20), APS plate (*n* = 13), or Mini + APS (*n* = 10). Demographic and injury variables were documented. We used X-ray radiography to determine plate fractures and screw dislocations after surgical fixation. *Results*: No statistical differences were noted in the demographic or injury variables. APS plates demonstrated fewer cases of plate fractures and screw dislocations than Mini plates (OR = 0.091, *p* = 0.008). *Conclusions*: The pre-contoured design of the APS plate demonstrated a superior rib implant failure rate as compared to the traditional Mini bone plate. Our study indicates that the APS plate may serve as an effective surgical tool for the treatment of flail chest.

## 1. Introduction

Flail chest is a common injury resulting from major trauma to the thoracic cage, occurring when more than three consecutive ribs have been broken. Flail chest could lead to mechanical instability and abnormal motion of the chest wall, potentially leading to chest deformity and cavity loss [1]. The resultant change in chest volume in flail chest cases commonly induces lung squeeze, dyspnea, and chronic pain [2,3]. 

In Taiwan, chest trauma is a common injury sustained in traffic accidents [4], with relevant literature reporting that flail chest occurs in approximately 7–10% of chest trauma cases [5,6]. In the past, non-surgical therapies have been the standard management techniques for flail chest, including intubation and internal pneumatic splint, pain management, pulmonary hygiene, and physiotherapy [7]. More recently, studies have revealed that the surgical stabilization of rib fractures may decrease the length of hospitalization, reduce chronic pain, and limit the potential disability of the chest wall due to progressive collapse of the flail segment. However, the fixation of rib fractures remains a challenge for surgeons due to the complex anatomy of the rib cage [8], as deficient contouring of plates to the rib surface may cause prominent discomfort or pain to patients; moreover, intraoperative contouring of plates to the rib surface is difficult, and the strength of the plates may be compromised by manual contouring [9,10].

In recent years, various three-dimensional pre-bent locking plates have been developed, which allow for the effective anatomic bony reconstruction of complex thoracic wall defects. The design of these anatomic plates is based on the natural contours of the ribs; as such, the pre-bent plates do not require the manual bending of a straight flat plate into a complex shape, and thereby produce an almost equivalent volume reconstruction. Additionally, the correct placement of the plates is challenging due to their length, the lack of available bony landmarks, and prolonged exposure of the rib cage during the procedure. To limit such surgical difficulties, patient-specific instruments (PSIs), such as surgical cutting guides or fixation guides, are designed to match the patient’s anatomy, while the plate fits the complex bone segments in the planned position, subsequently repairing the fractured segments [11,12]. Over the past decade, PSI technology has been applied in various orthopedic surgeries, including total joint arthroplasty and high tibia osteotomy. However, reports related to the application of PSIs for rib fixation are limited. In order to reduce intraoperative complications, and to simplify the surgical procedure, an implant system combined with a PSI for the fixation of rib fractures (APS Rib Fixation System, A PLUS BIOTECHNOLOGY Co., Ltd., Taiwan), which is based on anatomical rib data retrieved from the Asian population, has recently been developed.

This retrospective study analyzed our clinical data collected from cases using the APS Rib Fixation System for the stabilization of flail chest and compared it with the traditional Mini bone plate system. Data regarding healing outcomes, radiographies, and lengths of hospital stays were documented, analyzed, and compared.

## 2. Materials and Methods

From January 2020 to December 2021, 43 consecutive patients requiring surgical stabilization due to a flail chest injury were enrolled in the study. Indications for surgical stabilization were flail chest injury, with three or more consecutive fractured ribs. Exclusion criteria were age <21 years or >80 years, pregnant, severe closed-head injury, severe spinal cord injury, and associated extra-thoracic injuries that made survival during the follow-up period unlikely. The follow-up period of all patients spanned at least four months. The study protocol was approved by the Internal Review Boards of Show Chwan Memorial Hospital (project identification code: SCMH_IRB Number: 1101101; date of approval: 29 November 2021).

Patient demographics and injury details were obtained from pertinent medical records. The number and location of the rib fractures were extracted from three-dimensional computed tomography (CT) reconstructions. These pre-operative CT reconstructions were deemed essential to the planning of the surgical approach and stabilization procedure. 

Flail chest stabilization was performed through a video-assisted thoracoscopy. The video-assisted thoracoscopic surgery was performed to check for bleeding, to repair of lung parenchyma injury, and to precisely identify rib fracture sites. A mini thoracotomy was then performed to expose the rib fractures, with care taken to preserve the periosteum. The fractures were stabilized with anatomic plates and intramedullary rib splints. For plate fixation, anatomically correct pre-bent plates (APS Rib Fixation System) or manual bending plates (Mini bone plate) were cut to the desired length.

The plate fit was tested and corrected when necessary. The plates were attached to the fractured rib segments and temporarily stabilized with plate-holding forceps. A minimum of three self-tapping locking screws were inserted into each rib segment through the PSI. When possible, a long plate was used to bridge fractures of a flail rib segment. Upon flail chest stabilization, a chest tube and a layered muscular closure with absorbable suture were applied.

The surgical procedure was documented by noting the patient positioning, surgical approach, need for plate contouring, splint selection, and occurrence of intraoperative complications. The implant fixation was analyzed on radiographs to identify possible implant or fixation failure. 

Statistical analyses were performed, with mean ranges calculated for continuous variables, and compared using the Kruskal–Wallis test (a nonparametric alternative to the one-way ANOVA). Frequencies were calculated for categorical variables and compared using the Fisher exact test or Chi-square test when appropriate. A level of *p* < 0.05 was set as significant. All statistical analyses were performed using GraphPad Prism 9.2.0.

## 3. Results

**Figure 1:**
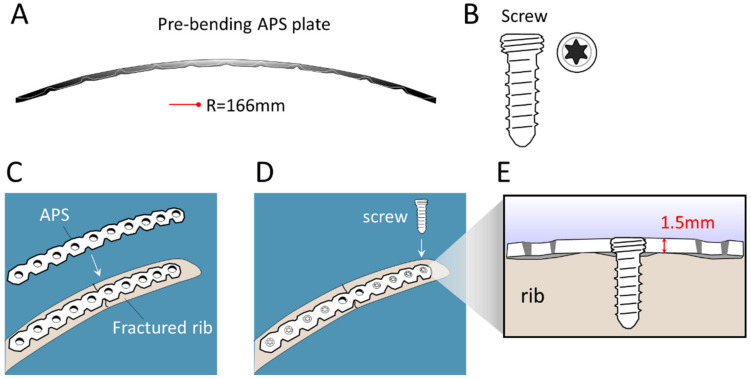
Schematic illustration of the configuration of the APS Rib Fixation System and its implantation in the scenario of repairing a rib fracture. (**A**) Sagittal view of APS plates shows a “pre-contoured” shape to fit an average rib shape, minimizing the need for intraoperative bending. R = radius. (**B**) The APS screw. (**C**,**D**) The procedure of implanting an APS plate on a bone injury. (**E**) The cross-sectional view of a screw-locked 1.5 mm thick APS plate.

**Figure 2:**
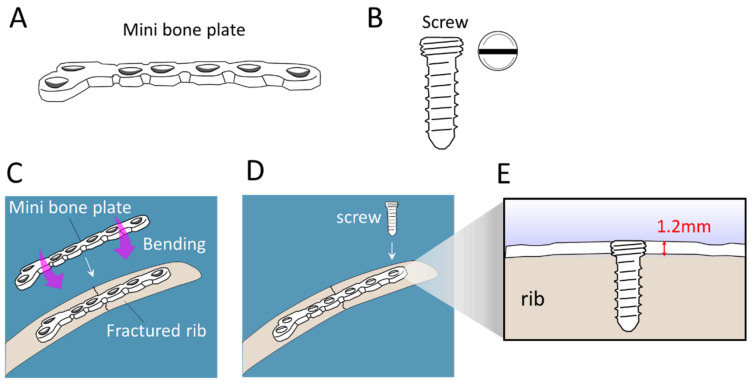
Schematic illustration of the configuration of a Mini bone plate and its implantation in the scenario of repairing a rib fracture. (**A**) General configuration of a Mini plate. (**B**) The Mini screw. (**C**,**D**) The Mini bone plate requires manual bending prior to implantation to a bone injury. (**D**) Screwing of the Mini plate. (**E**) The cross-sectional view of a screw-locked 1.2 mm thick Mini plate.

**Figure 3:**
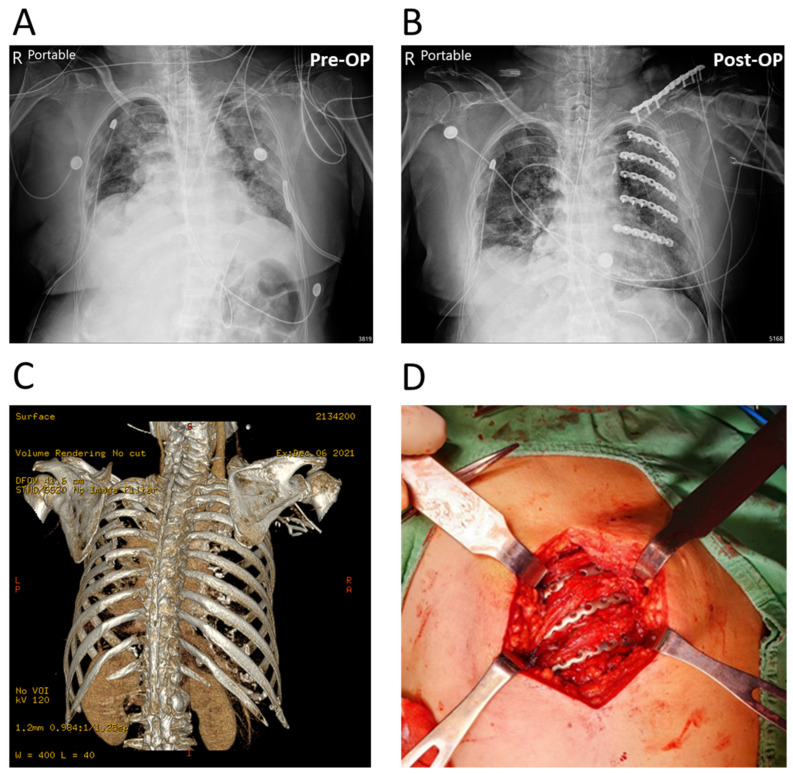
A representative case of multiple rib fractures repaired with APS plates. (**A**,**B**) AP radiograph of pre-operation (Pre-OP) and post-operation (Post-OP). (**C**) Three-dimensional CT reconstruction image of the injury. (**D**) Intraoperative photograph showing the rib-repairing technique with implanted APS plates.

The configurations of the APS Rib Fixation System and Mini bone plate are illustrated in Figure 1 and Figure 2, respectively. Notably, the “pre-contoured” structure of the APS plate (R = 166 mm) facilitated an optimized fit to the average rib shape, which minimized intraoperative bending (Figure 1). Meanwhile, the traditional Mini bone plate required manual bending prior to implantation (Figure 2). A representative case of the rib-repairing technique using the APS plates is shown in Figure 3.

Patient demographic data for both groups are shown in Supplementary Table S1. Patients were divided into groups according to the types of implanted plate, i.e., Mini, APS, and a combination of Mini and APS (Mini + APS). No statistical differences were noted in the demographic or injury variables. The lengths of hospital stays showed no significant difference (Supplementary Table S1). Three patients had post-surgical complications (Supplementary Table S2). One patient who received the Mini bone plate fixation required a debridement procedure due to postoperative seroma one month after the surgery. 

**Figure 4:**
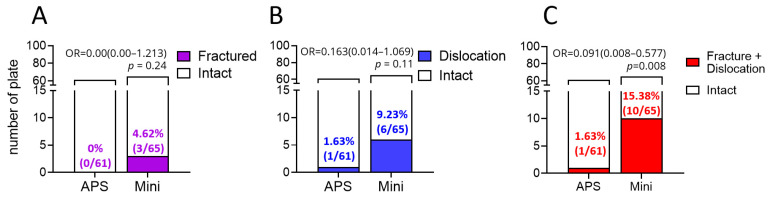
Comparison of fixation stability between the APS and Mini plates. The occurrences of (**A**) plate fracture, (**B**) screw dislocation, and (**C**) fracture plus dislocation. OR, odds ratio.

We evaluated the stability of the rib fixations by examining postoperative occurrences of plate fracture and screw dislocation. As shown in Figure 4, APS plates showed non-significant fewer cases of plate fracture (*p* = 0.24, Figure 4A) and screw dislocation (*p* = 0.11, Figure 4B). Of note, when combining cases of plate fracture and screw dislocation, the APS plates demonstrated statistically fewer cases than that of the Mini plates (*p* = 0.008, Figure 4C), indicating that APS plates present superior durability. Collectively, the pre-contoured APS plates demonstrated fewer plate fractures and screw dislocations as compared to the traditional Mini bone plates. Thus, APS plates may serve as an effective surgical tool for the treatment of flail chest.

## 4. Discussion

The most common cause of chest wall trauma is via direct impact to the chest region, such as that which may occur during a traffic accident or fall, while it is frequently seen in patients presenting to emergency departments. Flail chest occurs in approximately 15% of chest wall injuries [13]; furthermore, it is associated with a high morbidity rate [14] and a relatively high rate of mortality (approximately 16%) [6]. When flail chest cases are treated by a conservative option, fractured ribs may undergo progressive displacement during the healing process, leading to considerable deformity, volume loss, and chronic pain [9,15]. Hence, chest wall stabilization operations are commonly recommended, with studies indicating that this form of management offers multiple benefits, including shortened hospitalization times and duration of required mechanical ventilation, reduced ICU stay, decreased incidence of bone deformities, and fewer reports of chronic pain [16,17,18]. Meanwhile, the complex nature of the surgical procedure and subsequent implant failures are primary challenges related to the surgical stabilization procedure for flail chest [8,19]. The present study reports on cases of an implant system combined with PSI as applied in the surgical fixation of rib fractures, while the collected clinical results are compared with the traditional Mini bone plate system.

Our study identified no significant difference in hospital stay days between the two groups, although the average hospital stay for the APS group was indeed longer than that of the Mini group. This could be due to the longer incision required for the implant and PSI, which may necessitate a prolonged patient recovery time. Previous studies have reported that the average duration of a hospital stay for operative management patients ranges from 10 to 20 days [20,21], which is similar to our recorded data.

The patient follow-up data revealed a higher rate of implant failures in the Mini group as compared to the APS group, indicating that the pre-bent plates of the APS system could provide a more appropriate anatomical fit than the traditional plate. In addition, the pre-bent plates require less manipulation, leading to a reduced likelihood of metal fatigue. Moreover, the correct placement of these implants via the PSI technique may decrease residual stress on both the plates and screws, thereby reducing the risks of loosening screws and of structurally compromising the plates.

Due to the complex anatomy of the rib cage, the fixation of rib fractures remains a challenging procedure for surgeons. However, the PSI-based APS Rib Fixation System offers biomedical advantages for the repair of broken ribs. The pre-contoured design of the APS plates facilitates an optimized fit to the average rib anatomy, leading to reduced intraoperative complications and an overall simplification of the surgical procedure. In this study, the APS plates exhibited superior fixation stability as compared to the traditional Mini bone plate, thereby demonstrating their value as an effective surgical tool for the treatment of flail chest. Nonetheless, this study revealed no significant difference in the lengths of hospital stays for the two rib fixation systems. Of further note, several limitations to the present study exist due to the lack of data regarding patient pulmonary functions, post-surgical quality of life, and the recovery of working capacity; thus, further investigation that considers and assesses these factors is recommended. 

The superior performance of the APS plates lies in their durability and strength, which are required to support dynamic respiratory loading. To further understand the mechanistic and practical reliability factors, Bottlang et al. highlighted the indispensability of biochemical investigation [22]. For instance, Helzel et al. conducted a biomechanical study to evaluate the stability of a novel rib splint, wherein a prolonged, dynamic load was applied to human cadaveric ribs [23]. Although the retrospective design of the current study creates inherent limitations to documenting the biomechanical functionality of the rib implants, it is nevertheless a novel comparison of the APS plate and Mini bone plate systems in a clinical scenario and provides a foundation for future investigations. 

## 5. Conclusions

Taken together, the findings of this study indicate that the pre-contoured APS plate demonstrates superior performance as compared to the traditional Mini bone plate for the fixation of fractured ribs. The APS plate may thus serve as an effective surgical tool for the treatment of flail chest.

## Data Availability

The data used to support the findings of this study are available from the corresponding authors upon request.

## Supplementary Materials

The following supporting information can be downloaded at: https://www.mdpi.com/article/10.3390/medicina58030345/s1, Table S1: Demographic and Injury Data; Table S2: Post-surgical complications.
