# Supplementary material for: Improved Fixation Stability of a Dedicated Rib Fixation System in Flail Chest: A Retrospective Study
Source: Medicina (Kaunas). 2022 Feb 24;58(3):345. doi: 10.3390/medicina58030345 (PMC8955880; doi:10.3390/medicina58030345)
Supplement: Supplementary file 1 [file medicina-58-00345-s001.zip › medicina-1572280-supplementary.pdf]

Supplementary materials

Improved Fixation Stability of a Dedicated Rib Fixation System in Flail Chest: A Retrospective Study

Shang-Ting Tsai [1], Hung-Yu Lin [2], Chia-Ying Li [3,4,*] and Chih-Chien Lin [1,*]

[1] Department of Emergency Medicine, Show Chwan Memorial Hospital, Changhua 500, Taiwan; tim502323@gmail.com (S.-T.T.)

[2] Research Assistant Center, Show Chwan Memorial Hospital, Changhua 500, Taiwan; linhungyu700218@gmail.com (H.-Y.L.)

[3] Department of Surgery, Show Chwan Memorial Hospital, Changhua 500, Taiwan

[4] Graduate Institute of Biomedical Engineering, National Chung Hsing University, Taichung 402, Taiwan

* Correspondence: b86401115@ntu.edu.tw (C.-Y.L.); bkforever@gmail.com (C.-C.L.)

Table S1. Demographic and Injury Data

Variable	Mini (n = 20)	APS (n = 13)	Mini + APS (n = 10)	p value
Age (yr)	56.3 ± 17.2 (18-77)	62.6 ± 14.13 (38-89)	52.4 ± 23.9 (18-83)	0.39
Sex (male)	10 (50.0%)	7 (53.8%)	7 (70%)	0.57
Mechanism				
Motor vehicle collision	11 (55.0%)	10 (76.9%)	8 (80%)	0.26
Fall	9 (45.0%)	3 (23.1%)	2 (20%)	0.26
Other injury				
Clavicle facture	3 (15.0%)	3 (23.1%)	3 (30%)	0.61
Scapula facture	2 (10.0%)	3 (23.1%)	0 (0.0%)	0.22
Sternum facture	2 (10.0%)	0 (0.0%)	0 (0.0%)	0.29
Number of rib fractures	4.3 ± 2.9	3.8 ± 2.4	5.7 ± 2.6	0.26
Bilateral rib fractures	0 (0.0%)	1 (7.7%)	2 (20.0%)	0.12
Pneumothorax	8 (40.0%)	7 (53.8%)	7 (70.0%)	0.29
Hospital length of stay (d)	10.7 ± 7.0 (4-34)	19.6 ± 16.6 (5-59)	12.0 ± 7.6 (3-30)	0.31

Table S2. Post-surgical complications

Patient code	Treatment	Complications
R-1-1	APS	nil
R-1-2	APS	nil
R-1-3	APS	nil
MR-1-4	Mini+APS	nil
R-1-5	Mini+APS	nil
R-1-6	Mini+APS	nil
R-1-7	Mini+APS	nil
R-1-8	Mini+APS	nil
R-1-9	Mini+APS	nil
R-1-10	Mini+APS	nil
R-1-11	APS	nil
R-1-12	Mini+APS	nil
R-1-13	APS	nil
R-1-14	APS	ADM due to HEMATURIA
R-1-15	APS	nil
R-1-16	Mini+APS	nil
R-1-17	APS	nil
R-1-18	APS	ADM due to CONSCIOUS CHANGE
R-1-19	APS	nil
R-1-20	Mini+APS	nil
R-1-21	APS	nil
R-1-22	APS	nil
R-1-23	APS	nil
M-1-1	MINI	nil
M-1-2	MINI	nil
M-1-3	MINI	nil
M-1-4	MINI	nil
M-1-5	MINI	nil
M-1-6	MINI	nil
M-1-7	MINI	nil
M-1-8	MINI	nil
M-1-9	MINI	nil
M-1-11	MINI	nil
M-1-12	MINI	ADM due to seroma. Receiving debridement
M-1-13	MINI	nil
M-1-14	MINI	nil

M-1-15	MINI	nil
M-1-16	MINI	nil
M-1-17	MINI	nil
M-1-18	MINI	nil
M-1-19	MINI	nil
M-1-22	MINI	nil
M-1-23	MINI	nil
